# The Nordic Maintenance Care program: Effectiveness of chiropractic maintenance care versus symptom-guided treatment for recurrent and persistent low back pain—A pragmatic randomized controlled trial

**DOI:** 10.1371/journal.pone.0203029

**Published:** 2018-09-12

**Authors:** Andreas Eklund, Irene Jensen, Malin Lohela-Karlsson, Jan Hagberg, Charlotte Leboeuf-Yde, Alice Kongsted, Lennart Bodin, Iben Axén

**Affiliations:** 1 Karolinska Institutet, Institute of Environmental Medicine, Unit of Intervention and Implementation Research for Worker Health, Stockholm, Sweden; 2 Institute for Regional Health Research, University of Southern Denmark, Odense, Denmark; 3 Nordic Institute of Chiropractic and Clinical Biomechanics, Odense, Denmark; 4 Department of Sports Science and Clinical Biomechanics, University of Southern Denmark, Odense, Denmark; Baylor College of Medicine, UNITED STATES

## Abstract

**Background:**

For individuals with recurrent or persistent non-specific low back pain (LBP), exercise and exercise combined with education have been shown to be effective in preventing new episodes or in reducing the impact of the condition. Chiropractors have traditionally used Maintenance Care (MC), as secondary and tertiary prevention strategies. The aim of this trial was to investigate the effectiveness of MC on pain trajectories for patients with recurrent or persistent LBP.

**Method:**

This pragmatic, investigator-blinded, two arm randomized controlled trial included consecutive patients (18–65 years old) with non-specific LBP, who had an early favorable response to chiropractic care. After an initial course of treatment, eligible subjects were randomized to either MC or control (symptom-guided treatment). The primary outcome was total number of days with bothersome LBP during 52 weeks collected weekly with text-messages (SMS) and estimated by a GEE model.

**Results:**

Three hundred and twenty-eight subjects were randomly allocated to one of the two treatment groups. MC resulted in a reduction in the total number of days per week with bothersome LBP compared with symptom-guided treatment. During the 12 month study period, the MC group (n = 163, 3 dropouts) reported 12.8 (95% CI = 10.1, 15.5; p = <0.001) fewer days in total with bothersome LBP compared to the control group (n = 158, 4 dropouts) and received 1.7 (95% CI = 1.8, 2.1; p = <0.001) more treatments. Numbers presented are means. No serious adverse events were recorded.

**Conclusion:**

MC was more effective than symptom-guided treatment in reducing the total number of days over 52 weeks with bothersome non-specific LBP but it resulted in a higher number of treatments. For selected patients with recurrent or persistent non-specific LBP who respond well to an initial course of chiropractic care, MC should be considered an option for tertiary prevention.

## Introduction

Non-specific low back pain (LBP) is one of the most common and costly healthcare problems in society today [1]. The burden of disabling LBP on individuals, families, communities, industries and societies is substantial and is now the leading cause of activity limitation and work absence in the world [1, 2]. In Sweden (2012) 12% of the total cost of musculoskeletal disorders arises from spinal pain (ICD M50-M54) [3]. Given that LBP is often recurrent and has a large negative impact on society [4], it seems logical to focus on preventive strategies. In general, interventions aimed at prevention of chronic medical conditions are often described as either secondary or tertiary strategies.

Secondary prevention aims to reduce the impact of a condition (LBP) that has already manifested. This is usually done by encouraging strategies to prevent re-injury such as performing exercises. Tertiary prevention aims to reduce the impact of persistent or chronic LBP. This is usually done by helping people manage long-term, often complex pain conditions in order to improve their quality of life and ability to function.

The multifaceted etiology of LBP (including social, behavioral and psychological factors) implies that this is a complex problem in need of individually tailored interventions that are difficult to test experimentally [5]. To date, the number of LBP secondary or tertiary prevention strategies for which there is empirical evidence are few; there is moderate quality evidence that exercise combined with education reduces the risk of an episode of LBP [6].

Chiropractors are trained to assess and treat disorders of the musculoskeletal system, of which LBP is the most common [7, 8]. The majority of patients seeking chiropractic care receive some form of manual therapy, of which spinal manipulation and mobilization are the most common, often along with advice on exercise [7, 9–11]. Manual therapy has been shown to be effective for some patients with LBP [12, 13]. The outcome can be predicted by clinical history and demographic variables such as sex, social benefit, severity of pain, duration of continuous pain at first consultation, and additional neck pain [14, 15]. For patients receiving manual therapy, one of the strongest known predictors of a positive outcome is subjective improvement at the fourth visit. These patients, who are “fast responders”, also have a greater chance of a good outcome at three and 12 months [15].

It is common for chiropractors to recommend “maintenance care (MC)”, i.e. preventive consultations/visits for recurrent and persistent musculoskeletal pain and dysfunction [16]. MC can be viewed as a form of secondary or tertiary prevention and may include manual therapy, individual exercise programs and lifestyle advice delivered in regularly spaced visits over longer periods of time [9–11, 16–18].

Exactly how MC works is poorly understood but the main hypothesis is that treatment may improve biomechanical and neuromuscular function and address psychosocial issues, thereby reducing the risk of relapse into pain [19–24]. About one fifth of all visits to Scandinavian chiropractors are MC visits and 98% of Swedish chiropractors use the approach to some extent [16]. MC is traditionally employed as a long term-approach described as: *“…a regimen designed to provide for the patient’s continued well-being or for maintaining the optimum state of health while minimizing recurrences of the clinical status”* [25] *and “…treatment, either scheduled or elective, which occurred after optimum recorded benefit was reached, provided there was no evidence of relapse”* [26]. A number of studies in Scandinavia have investigated the indications, frequency and content of MC and there seems to be a common management strategy shared by chiropractors [11, 16–18, 27–33]. Although MC is widely used, the evidence of its effectiveness is equivocal [34, 35]. Two previous studies have investigated MC for LBP. Both contain methodological flaws because they did not take into account the current evidence about how MC is delivered in clinical practice. One of the studies was an efficacy study on a small sample [34]; the other was an RCT conducted in a hospital setting on a secondary care population with methods (frequency of visits and method of delivery) different from how chiropractors normally deliver MC [36]. The alternative to MC is usually to discontinue care and recommend the patients to schedule a new visit when they experience a new episode of pain or need treatment for other reasons. It is currently not known which method is most appropriate, MC or treatment only when there is perceived need by the patient and this is also debated within the chiropractic profession. A better understanding of the possible benefits of MC could greatly improve patient care by either changing clinical behavior by avoiding the procedure altogether or to recommend MC as a procedure to be used for selected patients.

We, therefore, designed and conducted a randomized controlled trial (RCT) which took the current state of evidence into account and tried to mimic the clinical decision-making process and approach of Scandinavian chiropractors today [37].

The aim of the present study was to investigate the effectiveness of MC as compared to chiropractic care given when there was a subject-perceived need (i.e. symptom-guided treatments) in a population of chiropractic patients with recurrent or persistent LBP.

The objectives of the study were to compare MC to symptom-guided care with regard to the total number of days with bothersome LBP over 52 weeks, the prevalence of days with pain per week over time as trajectories, and the total number of treatments.

## Materials and methods

### Trial design

This was a pragmatic, investigator- and assessor- blinded randomized controlled trial with a two arm parallel design. The trial is described in detail in a study protocol [37], and is briefly described below. No changes were made to the method after commencement; the trial has been conducted and analyzed according to the procedure described in the ethical application, approved by local ethics committee at Karolinska Institutet (2007/1458-31/4), which the published study protocol [37] is based on. The original protocol submitted for the ethical application along with ethical approvals have been included as supplementary material (S1 and S2 Files). The trial was registered in Clinical trials.gov; NCT01539863 (February 22, 2012). Funding bodies were the Institute for Chiropractic and Neuro-musculoskeletal Research, the European Chiropractors’ Union (project ID A13.02) and the Danish Chiropractic Research Foundation (grant number 11/148). None of the funding bodies have had any influence of the design, data collection, data analysis, interpretation of the data, or in the production of the manuscript.

### Participants

Consecutive patients with persistent or recurrent LBP were screened for eligibility in a stepwise manner (at Baseline 1, Baseline 2 and inclusion visit) as described in Table 1. “Baseline 1” was the initial screening visit, when patients first consulted the chiropractor. “Baseline 2” was the 4^th^ visit (or earlier depending on the patient’s subjective improvement), at which patients with a favorable response to treatment were identified. At this visit the patients were asked to rate their improvement on a 5-point Likert scale, ranging from “definitely worse” to “definitely improved”. Only patients who rated themselves as “definitely improved” were eligible to continue in the inclusion procedure. The study start occurred at this inclusion visit, when the initial treatment plan was completed and the clinician would recommend patients to either discontinue care or start a MC plan, i.e. when the clinician perceived that the patient’s next visit could be scheduled with an interval of 1 month or longer. This was the final step of the inclusion process, at which patients were randomly allocated to one of the treatment arms.

**Table 1:** Eligibility screening.

Time point	Inclusion criteria	Exclusion criteria
Baseline 1	Age 18–65 years. LBP with or without leg pain for more than 30 days during the past year. Previous episodes. Access to a mobile phone. Ability to send and receive SMS (text messages).	Pregnancy. Chiropractic treatment less than 3 months ago. Completely subsidized treatment from 3rd party payer. Serious pathology (i.e. acute trauma, cancer, infection, cauda equina, osteoporosis, vertebral fractures) or contraindications to manual therapy.
Baseline 2	Self-rated “definitely improved” by the 4th treatment.	
Study start	Interval between treatments is one month or more.	

**LBP**, non-specific low back pain (Table taken from study protocol, approved by authors [37]).

Patients were recruited between 2012 and 2016 from 40 Swedish chiropractors with clinics across Sweden. The clinicians were selected from an existing practice-based research network of chiropractors. Based on a previous survey [16], chiropractors who recommend MC to selected patients were identified and included in the study, while clinicians who never or always recommend MC were not included. This was to minimize bias from preconceptions and personal preferences about this treatment concept. Prior to enrolment in the study, all the clinicians attended a study procedure workshop. Detailed instructions regarding the protocol were discussed, and written information/instructions were provided. To ensure protocol fidelity, each participating clinician was contacted by a member of the project management group from Karolinska Institutet by weekly telephone calls until both parties were satisfied with the level of understanding of the treatment protocol and adherence to the project.

The study was approved by local ethics committee at Karolinska Institutet (2007/1458-31/4). All subjects signed an informed consent during the inclusion visit in the trial. The manuscript does not contain individual personal data, therefore consent for publication was not necessary.

### Interventions

The two treatment arms were MC (preventive treatment, i.e. clinician-controlled) and control (symptom-guided treatment, i.e. patient controlled). Both are strategies used in daily practice and are similar in nature, but they have different purposes and scheduling. The participating clinicians were instructed to tailor treatment-content and frequency of visits to each patient’s individual needs, in accordance with usual practice. In the MC group, the aim was to schedule patients before substantial pain reoccurred (i.e. controlled by the clinician), while in the control group patients were instructed to call in if and when the pain recurred (patient controlled). If patients in the control group made a new appointment, they were treated at one or several sessions until maximum benefit was reached and were once again instructed to call when in pain. If patients in the MC group experienced a new pain episode prior to the next scheduled visit, they were instructed to call for an earlier appointment and were cared for accordingly until they were ready to be scheduled for preventive visits again. MC visits were scheduled according to the clinicians’ judgement of patient need, but at intervals of no more than three months [32]. In order to achieve compliance with the treatment plan, patients in both groups paid half of the normal fee for these visits and the remaining half was donated by the clinician.

### Outcomes

The primary outcome for the trial was the number of days with bothersome LBP experienced during the study period (52 weeks), collected by means of weekly text messages using an automated system called “SMS-track” [38–40]. If a subject did not answer the SMS-question within 48 hours, a reminder message with the same question was sent. Weekly text messages provided information about the total number of days with bothersome LBP over the previous week. The single item question used in the SMS message was the following: “*On how many days during the past week were you bothered by your lower back (i*.*e*. *it affected your daily activities or routines)*? *Please answer with a number between 0 and 7”*. The question has been considered useful in previous studies in similar settings for measuring the clinical course of LBP [41–43].

‘Bothersomeness’ is a concept that has been used in several studies to measure the impact of pain rather than the actual presence of pain [44–47]. The term is thought to capture the presence of consequential pain and has been proposed as a standard outcome measure in LBP outcome research [46]. Bothersomeness has been found to correlate well with self-rated health [48], pain intensity [49], disability, prediction of work absence/healthcare consultations and psychological distress (anxiety, depression) [50]. In this study bothersomeness was used as a dichotomous outcome where the patient was asked to define whether each day with pain bothered them or not, i.e. affected daily activities or routines. This way, only pain that was relevant to the patient would be captured making each reported day with pain, at least theoretically, clinically relevant. The primary outcome used in this paper may be considered novel, but the psychometric properties have been tested in one study [49] where a positive correlation between pain intensity and number of days with bothersome LBP was shown.

At the first visit, patients were asked to complete a questionnaire with descriptive characteristics, pain intensity (0–10 Numeric Rating Scale) [51, 52] and self-rated health (EuroQol 5 dimensions) [53] as well as psychological and behavioral characteristics (MPI-S) [54–56]. At the fourth visit, another questionnaire was administered to record data about subjective improvement (recorded with a 5-point Likert scale from “definitely worse” to “definitely improved”), pain intensity (0–10 Numeric Rating Scale) [51, 52] and use of pain medication (Yes/No, type). At the study start visit, the final questionnaire in the inclusion process was administered in order to record further descriptive data, activity limitation (Roland Morris Disability Questionnaire) [57] and pain intensity (0–10 Numeric Rating Scale) [51, 52]. At 12 months’ follow-up, questionnaires were sent to patients so they could record self-rated health (EuroQol 5 dimensions) [53], activity limitation (Roland Morris Disability Questionnaire) [57], pain intensity (0–10 Numeric Rating Scale) [51, 52], treatment by other clinicians/practitioners/Medication (Yes/No, type), overall satisfaction with the care plan (recorded with a 5-point Likert scale from “definitely worth it” to “definitely not worth it”), overall health (recorded with a 5-point Likert scale from “perfect health” to “poor health”) [58], sick leave in the previous year (recorded as No, 1–7 days, 8–14 days, >15 days) [59] and perceived production loss due to pain (modified WPAI, LBP–V2-Swedish) [60]. At follow-up, the clinicians also received a questionnaire asking them to describe treatment content, side effects, number of visits/ dates by reviewing the patients’ clinical records retrospectively.

### Sample size

The sample size was estimated on the basis of a 30% difference in the number of days with bothersome LBP. The standard deviation from a previous sample [42] in a similar population was used to predict the variance in this sample for the sample size calculation. With a significance level of 5% it was estimated that 177 subjects were needed in each treatment arm to reach a power of 80%. To allow for dropouts a total of 400 subjects were aimed for in the recruitment to the trial.

### Randomization

A statistician created 40 permuted blocks with 10 subjects in each with an overall 1:1 allocation ratio between groups according to a randomization schedule. SPSS v20 was used to generate the randomization code. A research assistant created 400 consecutively numbered sealed opaque envelopes containing a letter with instructions and group assignment. At the pre-study workshops each clinician received 10 envelopes (one single randomization block) along with documentation of the study procedure. As patients became eligible to enter the study (third inclusion step) and consent of participation was given, the envelope was opened by the clinician in front of the subject in a consecutive sequence. The clinicians were instructed to describe the two treatment strategies to the patients as similar procedures, both in clinical use, without implying that either was more effective than the other. If a clinician was unable to recruit 10 subjects, the remaining envelopes were transferred to other clinicians.

### Blinding

The clinicians were blinded until the randomization procedure at the study start visit and had no opportunity to influence the assignment of the treatment arm. The investigators were blinded until the completion of the primary data analysis, when the group identities were revealed.

### Statistical methods

An intention to treat protocol was used and estimates were reported with arithmetic means and 95% confidence intervals (95% CI). To allow for accurate estimates for the primary outcome (number of days with bothersome LBP) collected by weekly SMS messages, individuals with ≥ 12 weeks of missing data were excluded from the analysis. No imputation of missing data was made.

The total number of days with bothersome LBP over 12 months was estimated using a Generalized Estimating Equations (GEE) linear regression model, using an independent correlation structure and a robust variance estimator. QIC-values (quasi-likelihood under the independence model criterion) were used to estimate the most appropriate correlation structure for the data. The analysis was performed in two steps with a primary analysis considering only group and time as covariates in the model and a secondary analysis considering also possible differences in baseline variables as covariates. These covariates were chosen for theoretical/logical reasons and thought to have a possible moderating effect on the outcome. Sex, treating clinician, pain intensity, self-rated health, activity limitation, patient expectations, presence of leg pain, type of work, use of analgesic medication, sick leave, number of treatments during inclusion period, and the number of days with bothersome LBP during the first week of the trial where considered possible covariates in the statistical model by including them individually and as interaction terms.

In the primary analysis of number of days with bothersome LBP, **time** and **group** as well as the interaction terms **time**^**2**^, group*time and group*time^**2**^ were significant (p<0.01), yielded the best goodness of fit with the data, and were therefore included in the final model.

In the secondary analysis of number of days with bothersome LBP, a stepwise exclusion procedure was used by always removing the least significant variable until a final model was reached where all variables left were significant (p<0.05) and the best goodness of fit value could be obtained. The final model in the secondary analysis included the variables **group**, **time**, **time**^**2**^, group*time and group*time^**2**^, **treating clinician**, **pain intensity** (at baseline), **use of analgesic medication** (during inclusion period), and **the number of days with bothersome LBP during the first week of the trial (Week 1)**.

The total number of visits was estimated with a GEE Poisson regression model, using an independent correlation structure and a robust variance estimator. The analysis of visit data followed a similar analytical strategy as the analysis of number of days with bothersome LBP with a primary analysis modeled with group and time and a secondary analysis also including the baseline variable as potential covariates. In the primary analysis the best model fit was achieved with the variables **group** and **time**^**2**^. The variables included in the secondary analysis were **Group**, **Time**^**2**^, **Activity Limitation** (RMDQ), **Use of analgesic medication** (during inclusion period), **Walking-standing type of work** and **Patient expectations**.

A sensitivity analysis, using a *per protocol* perspective, was also performed using both models form the primary and secondary analysis. This only included subjects in the MC group with four or more visits (subjects who had attended at least every third month).

The presence of pain over time as trajectories (number of days with bothersome LBP per week) is presented visually in graphs as crude mean weekly values as well as estimated weekly differences between groups (primary and secondary analysis) with 95% CI.

All analyses were performed using the statistical software SPSS version 25 and STATA version 12 [61, 62].

## Results

### Descriptive data

#### Participant flow

**Fig 1:**
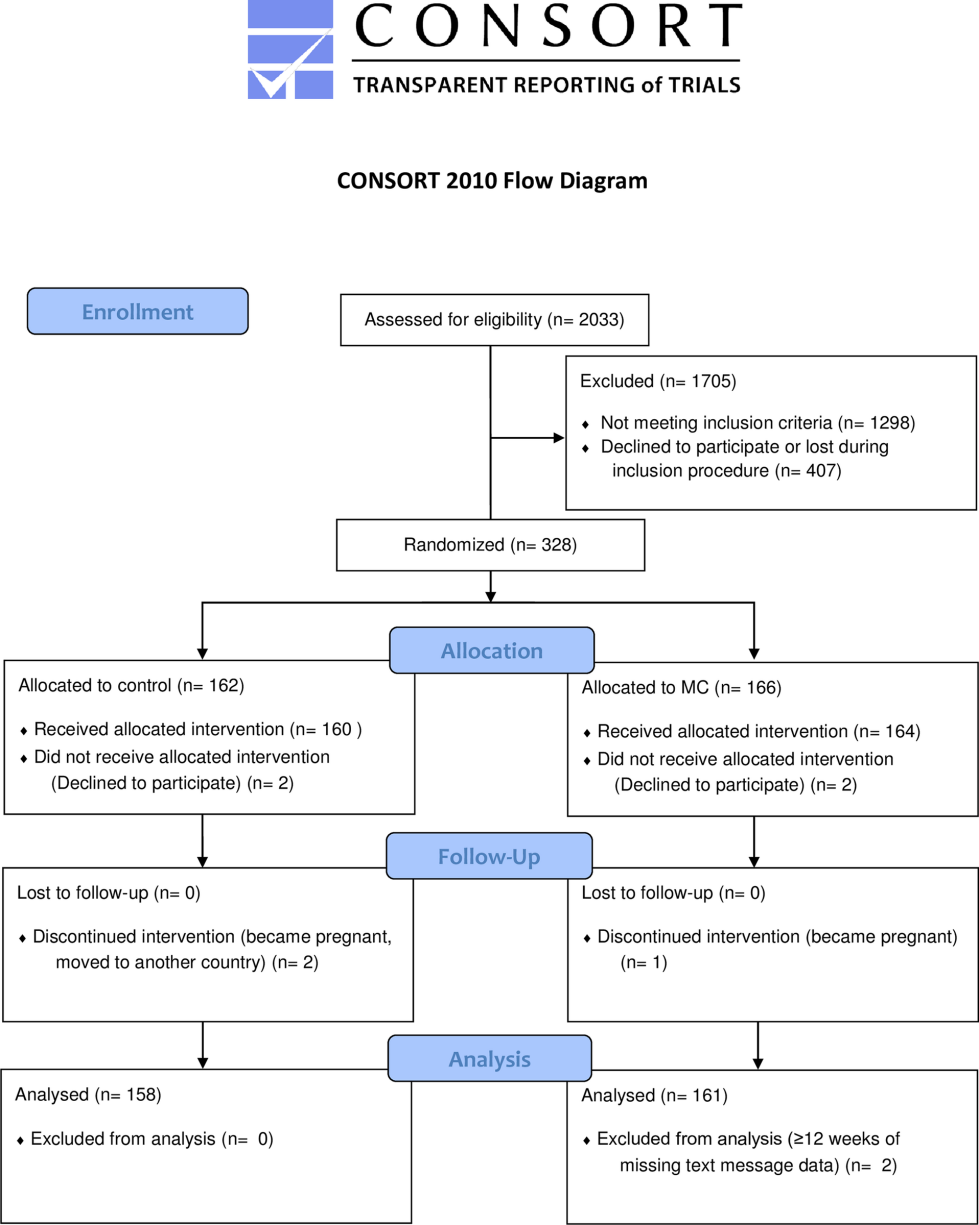
CONSORT 2010 Flow Diagram. MC, Maintenance Care.

The inclusion procedure and patient flow in the study are described in Fig 1.

#### Recruitment

The first subject was included in the study in April 2012 and the last in January 2016. A total of 2,033 patients were screened during their initial visit (baseline 1). Of these, 1,122 were not eligible (according to the inclusion and exclusion criteria described in Table 1). At the 4^th^ visit (baseline 2) a total of 616 subjects were screened for “definite improvement”. This resulted in a further 176 subjects being excluded from the trial (and an additional 295 subjects lost for unknown reasons). At the inclusion visit, 328 subjects were randomized into the trial (another 112 subjects were lost for unknown reasons). After randomization, seven subjects dropped out and could not be included in the data analysis (four changed their minds and did not want to participate; two became pregnant during the study period and one moved to another country). Of the 16,692 SMS messages that were sent during the study period, 1.1% were without a response. Two subjects were excluded from the data analysis due to 12 or more weeks of missing SMS data. The final data analysis was conducted in April 2017 and included 319 subjects.

The vast majority of subjects were recruited from middle to southern parts of Sweden, where the majority of the population lives. The areas bordering on the cities of Stockholm, Malmö, Karlstad, Falun and Luleå contributed the highest number of subjects in the trial. The distribution of subjects across Sweden has been illustrated as a heat-map based on the patients residential postal codes, see S1 Fig.

#### Clinicians

Out of the 40 recruited clinicians, five eventually chose not to participate due to the complexity of the inclusion process. Among the participating clinicians, the mean number of years in practice was 17.9, ranging from one to 38 years. The mean and median number of recruited subjects/clinician was 9.1 and 8.0, ranging from one to 42. These clinicians are part of a practice-based research network and have been found to be a good representation of the chiropractors of the Swedish Chiropractic Association (LKR) in previous studies [42, 63–66]).

#### Baseline data

A detailed description of the baseline demographic and clinical characteristics of the subjects during all steps of the inclusion process is provided in a supplementary table, S1 Table. Overall, the subjects were similar at the different steps of the inclusion process and differed only in the criteria on which the inclusion process was based. No systematic differences can be observed among the individuals who were lost during the inclusion procedure compared to the individuals who were followed up in the trial.

A detailed description of the baseline demographic and clinical characteristics of the subjects in both groups who completed the trial is presented in Table 2. The two groups had similar descriptive baseline data, which indicates that the randomization had worked well.

**Table 2:** Baseline data for control and MC groups, n = 324.

Variable		Control, n = 160	MC, n = 164	p-value
**1**^**st**^ **visit**				
Pain in the thigh, % (n)		22.8 (36)	20.9 (34)	0.719 ^A^
Pain in the thigh and lower leg, % (n)		17.7 (28)	21.5 (35)	0.443 ^A^
Pain in the lower leg, % (n)		2.5 (4)	3.7 (6)	0.540 ^A^
Never visited chiropractor for this problem before, % (n)		50.0 (79)	46.6 (76)	0.253 ^A^
Pain in the neck and/or thoracic spine n = 269, % (n)		66.9 (87)	69.1 (96)	0.820 ^A^
Pays for treatment n = 298, % (n)	Completely by patient	93.2 (136)	88.8 (135)	0.394 ^A^
	Partly by other	6.8 (10)	11.2 (17)	0.239 ^A^
Patients believe that their pain will get better 0–10 (No chance—Very likely), mean (SD)		8.1 (2.0)	8.4 (1.7)	0.134^B^
Lives alone, % (n)		13.9 (22)	13.5 (22)	0.946 ^A^
MPI cluster ID / DYS / AC, % (n)		23.8/39.3/36.9 (122)	25.0/38.6/36.4 (132)	0.946 ^A^
Pain severity (MPI) 0–6, mean (SD)		3.3 (1.2)	3.3 (1.1)	0.595 ^B^
Interference (MPI) 0–6, mean (SD)		2.7 (1.4)	2.9 (1.3)	0.254 ^B^
Life Control (MPI) 0–6, mean (SD)		3.6 (1.1)	3.4 (1.1)	0.361 ^B^
Affective distress (MPI) 0–6, mean (SD)		2.6 (1.4)	2.7 (1.3)	0.328 ^B^
Support (MPI) 0–6, mean (SD)		3.8 (1.7)	4.1 (1.6)	0.190 ^B^
Punishing responses (MPI) 0–6, mean (SD)		1.0 (1.2)	1.1 (1.3)	0.532 ^B^
Solicitous responses (MPI) 0–6, mean (SD)		2.6 (1.4)	2.7 (1.4)	0.812 ^B^
Distracting responses (MPI) 0–6, mean (SD)		2.8 (1.4)	2.8 (1.4)	0.978 ^B^
Pain intensity at 1st visit (first measure) 0–10, mean (SD)		5.3 (2.1)	5.2 (2.1)	0.741 ^B^
EQ5D score baseline, mean (SD)		0.71 (0.19)	0.68 (0.22)	0.252 ^B^
Health in general (study start), % (n)	Excellent	6.3 (10)	4.3 (7)	0.728 ^A^
	Very good	21.9 (46)	32.5 (53)	
	Good	42.4 (67)	37.4 (61)	
	Quite poor	11.4 (18)	16.0 (26)	
	Poor	3.8 (6)	3.1 (5)	
**4**^**th**^ **visit**				
Chiropractor believes that MC is appropriate for patient, % (n)		98.5 (130)	97.9 (137)	0.688 ^A^
Has taken analgesic medication for the pain, % (n)		15.0 (22)	19.9 (30)	0.366 ^A^
Pain intensity at 4th visit (second measure) 0–10, mean (SD)		2.4 (1.7)	2.5 (1.7)	0.624 ^B^
**Study start**				
Number of days between 1^st^ visit and study start, mean (SD)		53.4 (9.4)	46.9 (30.5)	0.148 ^B^
Age at study start, mean (SD)		43.0 (13.1)	43.4 (11.7)	0.707 ^B^
Female, % (n)		60.3 (85)	64.0 (96)	0.572 ^A^
Physically heavy type of work ^D^, % (n)		12.7 (20)	9.2 (15)	0.340 ^A^
Intermittent heavy/light type of work ^D^, % (n)		31.0 (49)	31.9 (52)	0.805 ^A^
Walking/standing type of work ^D^, % (n)		28.5 (45)	35.0 (57)	0.226 ^A^
Sitting type of work ^D^, % (n)		43.7 (69)	48.5 (79)	0.456 ^A^
Number of treatments during initial period, mean (SD)		6.1 (2.6)	5.8 (2.4)	0.205 ^B^
Type of treatment during inclusion process, % (n)	SMT/MOB/ACT/DROP	87.3 (138)	91.4 (149)	0.252 ^A^
	STT	63.3 (100)	65.6 (107)	0.634 ^A^
	Information/advice	69.0 (109)	77.3 (126)	0.106 ^A^
	Other			0.266 ^A^
Sick leave during the past year (at study start), % (n)	No sick leave	86.2 (119)	89.7 (129)	0.032 ^A^
	1–7 days	5.1 (7)	8.3 (12)	
	8–14 days	3.6 (5)	0.7 (1)	
	>15 days	5.1 (7)	1.4 (2)	
Pain intensity at study start 0–10, mean (SD)		2.2 (1.8)	2.1 (1.6)	0.375 ^B^
Week 1, number of days with pain, n = 309, mean (SD)		2.3 (2.0)	2.5 (2.0)	0.265^A^
RMDQ Score (study start), n = 306, mean (SD)		4.7 (4.0)	5.0 (4.0)	0.664 ^B^

MC, Maintenance Care; SD, Standard Deviation; MPI, West-Haven Yale Multidimensional Pain Inventory; AC, Adaptive Coper; ID, Interpersonally Distressed; DYS, Dysfunctional; SMT, spinal manipulative therapy; MOB, Mobilization; ACT, Mechanically assisted spinal manipulative therapy using the activator instrument; DROP, Mechanically assisted spinal manipulative therapy using a drop mechanism in table; STT, soft tissue treatment; ATM, use of ATM treatment table; RMDQ, Roland Morris Disability Questionnaire; EQ5D, EuroQol 5 dimensions;

^D^ Possible to have multiple answers on type of work (proportion of total in group);

SD, Standard Deviation;

^A^, Chi Square test;

^B^, One-Way ANOVA.

#### Follow-up data at 52 weeks

Table 3 presents the follow-up data collected at the end of the study period (12 months).

**Table 3:** Follow up data at 52 weeks.

Variable		Control, n = 138	MC, n = 152	p-value
Health in general (follow up), n = 289, % (n)	Excellent	12.3 (17)	12.6 (19)	0.890 ^A^
	Very good	44.2 (61)	43.7 (66)	
	Good	34.1 (47)	35.1 (53)	
	Quite poor	8.7 (12)	8.6 (13)	
	Poor	0.7 (1)	0.0 (0)	
Has received treatment from other healthcare professional during study period (at follow up), n = 289, % (n)		39.4 (54)	32.2 (49)	0.203 ^A^
Care plan is worth continuing with (at follow up), n = 288, % (n)	Definitely	47.8 (65)	59.2 (90)	0.092 ^A^
	Possibly	26.5 (36)	25.7 (39)	
	Either/or	14.7 (20)	9.2 (14)	
	Hardly	6.6 (9)	5.3 (8)	
	Definitely not	4.4 (6)	0.7 (1)	
Sick leave during study period (at follow up), n = 289, % (n)	No sick leave	83.3 (115)	85.4 (129)	0.430 ^A^
	1–7 days	10.1 (14)	9.9 (15)	
	8–14 days	4.3 (6)	1.3 (2)	
	>15 days	2.2 (3)	3.3 (5)	
Type of treatment, during study period, multiple answers possible, n = 319, % (n)	SMT	85.5 (118)	94.1 (143)	0.001 ^A^
	MOB/ACT/DROP	29.7 (41)	34.2 (52)	0.212 ^A^
	STT	61.6 (85)	63.2 (96)	0.293 ^A^
	ATM	8.7 (12)	12.5 (19)	0.205 ^A^
	Information/advice	62.3 (86)	75.0 (114)	0.002 ^A^
	Other	36.2 (50)	32.2 (49)	0.815 ^A^
Side effects of treatment during study period, multiple answers possible, n = 319, % (n)	Local soreness	21.0 (29)	22.4 (34)	0.535 ^A^
	Felt tired	3.6 (5)	2.6 (4)	0.714 ^A^
	New radiating pain	0	0	-
	Other	4.3 (6)	3.3 (5)	0.735 ^A^
LBP effect on productivity during past month 0–10 (Did not affect work—prevented work completely), at follow up, n = 287, mean (SD)		2.0 (2.3)	1.7 (1.9)	0.239 ^B^
Pain intensity at follow up visit 0–10, n = 288, mean (SD)		2.0 (2.1)	1.9 (2.0)	0.878 ^B^
EQ5D score follow up, n = 273 mean (SD)		0.84 (0.14)	0.85 (0.12)	0.553 ^B^
RMDQ Score (follow up), n = 248, mean (SD)		3.6 (4.3)	3.4 (3.6)	0.163 ^B^

MC, Maintenance Care; SD, Standard Deviation; SMT, spinal manipulative therapy; MOB, Mobilization; ACT, Mechanically assisted spinal manipulative therapy using the activator instrument; DROP, Mechanically assisted spinal manipulative therapy using a drop mechanism in table; STT, soft tissue treatment; ATM, use of ATM treatment table; RMDQ, Roland Morris Disability Questionnaire; EQ5D, EuroQol 5 dimensions; SEM, Standard Error of the Mean;

^A^, Chi Square test;

^B^, One-Way ANOVA.

### Outcomes

#### The total number of days with bothersome LBP over 52 weeks

The total number of days with bothersome LBP (primary analysis) over the twelve months was 85.2 (95% CI = 83.5, 87.0) for the MC group and 98.0 (95% CI = 95.9, 100.1) for the control group. In the sensitivity analysis, using a *per protocol* perspective, only subjects who had four or more visits in the MC group were included (n = 278). In the sensitivity analysis (per protocol) the total number of days with bothersome LBP (primary analysis) over the twelve months was 89.1 (95% CI = 87.0, 91.07) for the MC group and 98.0 (95% CI = 95.9, 100.1) for the control group. Outliers were considered as part of the primary analysis but did not change the interpretation or the estimate substantially. Group differences from the primary and secondary analysis are reported in Table 4.

**Table 4:** Difference between groups in total number of days with bothersome LBP and visits during study period (Control-MC).

Model	Intention to treat analysis (n = 319)	Per protocol analysis (n = 278)
**Difference in days with bothersome LBP (95% CI)**		
Primary analysis ^A^	12.8 (10.1, 15.5)*	9.0 (6.1, 11.9)*
Secondary analysis ^B^	13.9 (11.7, 16.0)*	12.6 (10.2, 14.9)*
**Difference in number of visits (95% CI)**		
Primary analysis ^C^	-1.7 (-2.1, -1.8)*	-3.8 (-3.9, -3.6)*
Secondary analysis ^D^	-1.6 (-1.8, -1.5)*	-3.4 (-3.5, -3.2)*

MC, Maintenance Care; 95% CI, 95% Confidence Intervals; SEM, Standard Error of the Mean,

*, p-value = <0.001;

^A^, Variables in GEE model: Group, Time, Time^2^, Group*Time, Group*Time^2^;

^B^, Variables in GEE model: Group, Time, Time^2^, Group*Time, Group*Time^2^, Number of days with pain week 1 of study period, Clinician, Pain intensity at baseline, Use of analgesic medication during inclusion period;

^C^, Variables in GEE model: Group, Time^2^;

^D^, Variables in GEE model: Group, Time^2^, Activity Limitation (RMDQ), Use of analgesic medication during inclusion period, Walking-standing type of work, Patient expectations.

#### The development of pain over time as trajectories over 52 weeks

**Fig 2:**
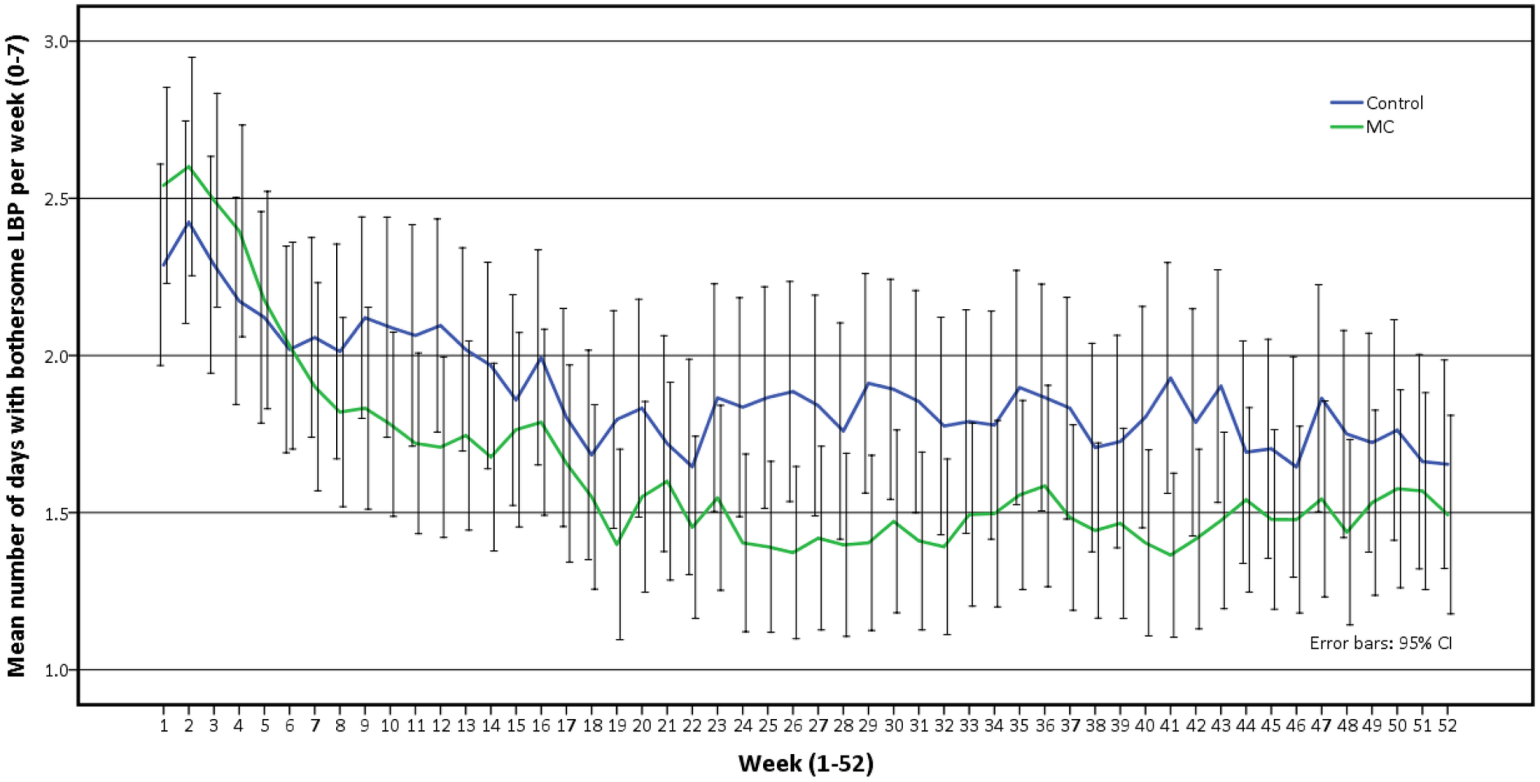
Mean number of days with bothersome LBP per week, observed data. LBP, Non-specific Low Back Pain; MC, Maintenance Care; 95% CI, 95% Confidence Interval.

**Fig 3:**
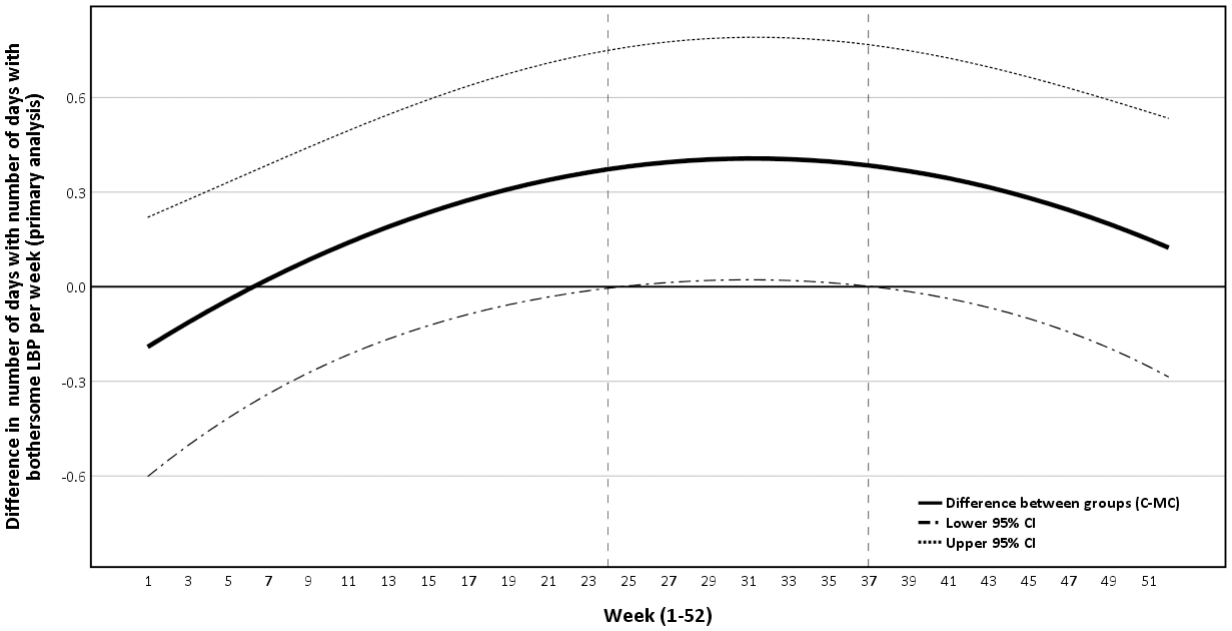
Mean difference in number of days with bothersome LBP per week, primary analysis. Variables in GEE model: Group, Time, Time^2^, Group*Time, Group*Time^2^; the difference is statistically significant (at 5% level) between week 24 to 37; LBP, Non-specific Low Back Pain; MC, Maintenance Care; C, Control; 95% CI, 95% Confidence Interval.

**Fig 4:**
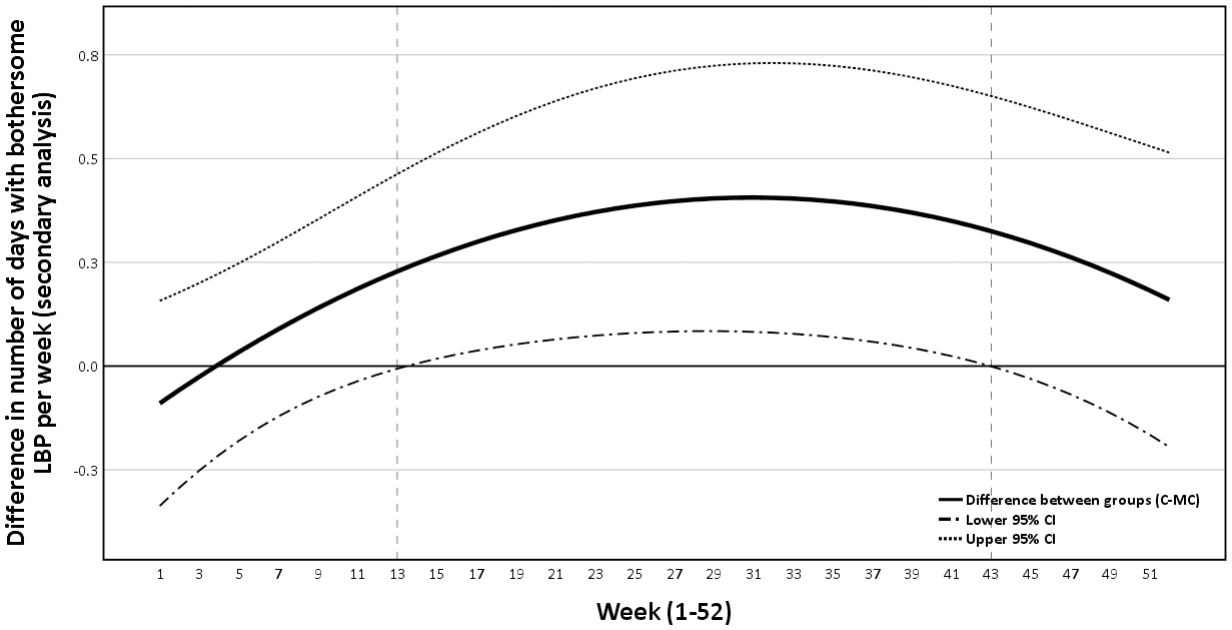
Mean difference in number of days with bothersome LBP per week, secondary analysis. Variables in GEE model: Group, Time, Time^2^, Group*Time, Group*Time^2^, Number of days with pain week 1 of study period, Clinician, Pain intensity at baseline, Use of analgesic medication during inclusion period; the difference is statistically significant (at 5% level) between week 13 to 43; LBP, Non-specific Low Back Pain; MC, Maintenance Care; C, Control; 95% CI, 95% Confidence Interval.

The crude pain trajectory of mean number of days with bothersome LBP per week is illustrated in Fig 2. Data shows that both groups continued to improve during the first quarter of the study period and then appeared to stabilize. Furthermore, the development over time for the two groups was different at the beginning of the study period, with the MC group having a faster reduction in days with bothersome LBP and reaching a lower steady state earlier. Figs 3 and 4 illustrates the pain trajectory for the difference in mean number of days with bothersome LBP per week modeled according to the primary and secondary analysis.

#### Visits

The total number of visits (primary analysis) over the twelve months was 6.7 (95% CI = 6.6, 6.8) for the MC group and 4.8 (95% CI = 4.7, 4.9) for the control group. In the sensitivity analysis (per protocol) the total number of visits (primary analysis) over the twelve months was 8.6 (95% CI = 8.5, 8.7) for the MC group and 4.8 (95% CI = 4.7, 4.9) for the control group. Outliers were considered as part of the primary analysis but did not change the interpretation or the estimate substantially. Group differences from the primary and secondary analysis are reported in Table 4. The most common treatment was spinal manipulation (85.5%, 94.2%), followed by information/advice (62.3%, 75.0%) and soft tissue treatment (61.6%, 63.2%) for the control and MC groups respectively. The proportions of the different treatments in each group are shown in Table 3.

#### Harm

No serious harm was reported by either of the two treatment groups, although some minor side effects were reported. The proportions of common side effects are described in Table 3. See Table 5 for a full description of side effects reported by the clinician retrospectively from the clinical records.

**Table 5:** Other specific treatment side effects, reported qualitatively.

Type of side effect	n	Duration
**Control**		
Headache	3	1 day
Nausea	1	1 day
Bruise over muscle	1	-
Coxygodynia	1	3 days
**MC**		
Increased intensity of low back pain	1	2–3 days
Lumbar stiffness	2	1–2 days
Nausea	1	1 day
Feeling of restlessness	1	1 day

MC, Maintenance Care.

## Discussion

This is one of the first studies to test the effect of preventive manual care performed by chiropractors (Maintenance Care) for recurrent and persistent LBP. The pragmatic nature of this randomized clinical trial, which uses all current evidence in the field and mimics clinical practice, makes this a unique contribution to the field of manual care and primary care medicine. MC resulted in a reduction in the total number of days per week with bothersome LBP compared with symptom-guided treatment. This reduction may be important for patients, as it was observed steadily over a period of 12 months, accumulating to a sum almost equal to the total number of work days in a month. Furthermore, the MC group required only a slightly higher number of visits to the chiropractor than the control group.

The secondary analysis of the data which included baseline variables as covariates did not change the estimates substantially. Although the difference in number of days is modest, the costs in terms of increased number of visits is small and the results should be considered of relevance for patients, clinicians and policymakers.

Where overall pain trajectories are concerned, both groups continued to improve after inclusion in the trial. This could be an indication that the groups had not achieved maximum benefit from the initial treatment when they were randomized into the trial. However, of the two groups the MC improved faster and achieved the steady state phase earlier with a lower mean number of days with LBP per week. The difference between the groups holds for the rest of the study period but becomes smaller towards the end. It is important to point out that from our data we cannot extrapolate the outcome beyond 12 months if patients were to continue with MC.

The main strength of this trial is the randomized design and the large longitudinal data set of high quality. The data set with 16,505 data points and 1.1% missing data is in itself remarkable and allows for a detailed analysis of the patients’ clinical course over 12 months.

The study sample was purposely chosen according to specific evidence-based criteria to mimic clinical practice. Individuals were selected on the basis of their need for preventive interventions because of the recurrent and persistent nature of their condition. However, only individuals who responded well to an initial course of care were included [15, 67]. By selecting patients with the most favorable response to treatment, the intervention targeted the most relevant patients where the potential benefit was believed to be the greatest. Previous research has shown that this process of stratification is how Scandinavian chiropractors select suitable patients for MC [16, 27–30, 33]. The results are therefore easily generalizable to current clinical practice by chiropractors in the Scandinavian countries and can be easily transferred and implemented.

Another strength of the study is the multicenter design featuring many clinics that recruited subjects in a geographical distribution that resembled the distribution of the general population of Sweden.

The treatment was not reported as being linked to any serious harm and both the intervention and the control regimes must be considered safe treatments. There were minor transient reactions to the treatment, evenly distributed between the two groups, such as local soreness for 1–2 days. Transient reactions such as increased stiffness and pain are common and have been reported in previous trials and are considered normal reactions to this type of treatment [67, 68]. A few patients also reported uncommon but transient (1–3 days) reactions such as headache, nausea, restlessness and coxygodynia.

The results of this study support the findings of the only other sufficiently powered RCT, by Senna and Shereen, to have investigated preventive manual care [36]. They found that patients who continued to receive spinal manipulation after an initial course of care had lower pain and disability scores at a 10-month follow-up. However, there were major differences in the inclusion procedure and the application of the treatment compared with the present trial. In fact, the treatment protocol used in the trial by Senna and Shereen differs greatly from the current clinical practice of MC that has been described in the literature. Nevertheless, it does indicate that these types of patients benefit from continued care, perhaps regardless of the type of manual care provided.

Whether the effect observed in this trial is clinically relevant is a different question altogether, as there is no previous data about what constitutes a minimally clinically-important difference with regards to change scores or absolute levels related to days with bothersome pain. The very nature of our measurement was, in itself, designed to be clinically relevant, because patients were asked to judge the impact of pain and whether it bothered them, i.e. whether the consequences of pain were relevant to them. The estimates are therefore likely to have clinical relevance for the patient, particularly when we look at the entire 12 month period. However, as the weekly difference is small it is possible that patients might not rate the difference as clinically meaningful on a week to week basis.

A weakness of the trial was the fairly large number of individuals who were lost during the 3 step inclusion process. Of the subjects who were eligible after the first visit, 32% were lost and of the subjects who were eligible at the fourth visit, 25% were lost. The reasons for these losses are unknown to the research team, however one likely explanation is that a very fast or a very poor improvement could have made the patient decide to discontinue care. Given the complex nature of the inclusion procedure, it is also possible that some clinicians forgot to administer the follow-up forms. The baseline data for the lost subjects were very similar to those for the included and the excluded subjects, which indicates a non-systematic error and therefore not a major concern. However, this drop-out rate might well mirror situations in real clinical practice, where many people stop treatment regardless of the advice given by the clinician.

We also failed to include the minimum number of subjects estimated by the power analysis. However, the power analysis was challenging given that MC is a poorly investigated procedure using a novel instrument (days with bothersome pain) with little robust prior data to base it on. This should be taken into consideration when interpreting the results.

Some of the data-collecting clinicians raised concerns about the strict fourth visit eligibility criteria and argued that there were a number of patients who reported a definite improvement at later visits and therefore might have been suitable candidates for the trial. Concerns were also raised about the intervals at which the clinicians were instructed to schedule preventive visits (between 1 and 3 months). Some clinicians suggested that some patients (the ones with more persistent pain) would have benefited from a shorter intervals than 1 month to be able to prevent future episodes. It should be noted that both of these requirements were enforced due to previous research results. In a controlled experiment such as this, it is impossible to completely mimic all the individual variations of clinical reality. Concerns such as these are therefore valid but difficult to act on.

Future work will look at how the intervention and control groups differ in a secondary analysis focusing on the development of pain around the time of the visits. The higher number of visits in the MC group represents a higher use of resources and the achieved effect should be considered in the light of this. Cost-effectiveness and the cost-utility of the intervention will therefore be explored in a coming study.

## Conclusion

In patients with recurrent and persistent LBP who responds well to an initial course of manual therapy, MC resulted in a reduction in number of days with bothersome LBP per week, compared with symptom-guided treatment. In total, the MC group had on average 12.8 fewer days with bothersome LBP over 12 months. The effect of the intervention was achieved at the cost of 1.7 additional visits to the chiropractor. For patients with recurrent and persistent LBP who are selected according to evidence-based criteria, MC should be considered as an option for tertiary prevention.

## Supporting information

S1 File Original trial protocol part of the ethical application. (PDF)

S2 File English translation of the key methodological portions of the original trial protocol part of the ethical application. (PDF)

S3 File CONSORT 2010 checklist of information to include when reporting a randomized trial. (PDF)

S1 Fig Heat map illustrating the density of number of requited subjects in the trial geographically across Sweden. Red zones represents the highest numbers of requited subjects followed by yellow, green and blue.
(TIF)

S1 Table Descriptive patient data at the different stages of the inclusion process. LBP, Low Back Pain; ^A^, Inclusion criteria; ^B^, Exclusion criteria; MPI, West-Haven Yale Multidimensional Pain Inventory; AC, Adaptive Coper; ID, Interpersonally Distressed; DYS, Dysfunctional; MC, Chiropractic Maintenance Care; RMDQ, Roland Morris Disability Questionnaire; EQ5D, EuroQol 5 dimensions; SD, Standard Deviation.
(PDF)

## Abbreviations

95% CI: 95% Confidence Intervals
AC: Adaptive Coper
ACT: Mechanically assisted spinal manipulative therapy using the activator instrument
DROP: Mechanically assisted spinal manipulative therapy using a drop mechanism in table
DYS: Dysfunctional
EQ5D: EuroQol 5 dimensions
GEE: Generalized Estimating Equations
ID: Interpersonally Distressed
LBP: Low Back Pain
LKR: Swedish Chiropractic association
MC: Maintenance Care
MOB: Mobilization
MPI: West-Haven Yale Multidimensional Pain Inventory
p: p-value
QIC: Quasi-Likelihood Criterion
RCT: Randomized Controlled Trial
RMDQ: Roland Morris Disability Questionnaire
SD: Standard Deviation
SMS: Text Message
SMT: Spinal Manipulative Therapy
STT: Soft Tissue Therapy
