# Supplementary material for: The Nordic Maintenance Care program: Effectiveness of chiropractic maintenance care versus symptom-guided treatment for recurrent and persistent low back pain—A pragmatic randomized controlled trial
Source: PLoS One. 2018 Sep 12;13(9):e0203029. doi: 10.1371/journal.pone.0203029 (PMC6135505; doi:10.1371/journal.pone.0203029)
Supplement: S1 File — (PDF) [file pone.0203029.s001.pdf]

ANSÖKAN OM ETIKPRÖVNING

se anvisning sid. 16

Uppgifter som fylls i av den regionala etikprövningsnämnden

Ankomstdatum: Dnr:

Avgift inbetald datum: Begäran om komplettering av ansökan:

Ansökan komplett:

Begärd information inkommen: Begäran om ytterligare information:

Expeditionsdatum: Beslutsdatum:

Uppgifter som fylls i av sökanden

Till Regionala etikprövningsnämnden i: Stockholm
(Den regionala etikprövningsnämnd till vars upptagningsområde forskningshuvudmannen hör, se www.forskningsetikprovning.se)

Projekt

Ange en beskrivande titel på svenska för lekmän, utan sekretesskyddad information.<br>Ange också i förekommande fall projektets identitet, projektets/forskningsplanens (protokollets eller prövningsplanens) nummer, version, datum osv.

Kiropraktisk behandling vid återkommande ländryggssmärta.

Projektnummer/identitet: Version nummer:

Ansökan avser (gäller även vid begäran om rådgivande yttrande):

forskning där endast en forskningshuvudman deltar (5000 kr) ☒

forskning där fler än en huvudman deltar (16000 kr) ☐

forskning där mer än en forskningshuvudman deltar, men där samtliga forskningspersoner eller forskningsobjekt enligt 4 § lagen (2003:460) om etikprövning av forskning som avser människor, har ett omedelbart samband endast med en av forskningshuvudmännen (5000 kr) ☐

endast behandling av personuppgifter (5000 kr) ☐

forskning som gäller klinisk läkemedelsprövning (16000 kr) ☐

ändring av tidigare godkänd ansökan (enligt 4 §) (2000 kr) ☐

Om nämnden finner att studien/forskningsprojektet inte faller inom lagens för etikprövning tillämpningsområde önskas ett rådgivande yttrande

Ja: ☒ Nej: ☐

1. Information om forskningshuvudman m.m.

1:1 Sökande forskningshuvudman

Ansökan om etikprövning av forskning skall göras av forskningshuvudmannen. *Med forskningshuvudman avses en statlig myndighet eller en fysisk eller juridisk person i vars verksamhet forskningen utförs.* Inom staten utförs forskning främst vid lärosätena, men även vid vissa andra myndigheter, som t.ex. Brottsförebyggande rådet och Socialstyrelsen. Kommuner och landsting kan vara forskningshuvudmän, liksom privaträttsliga juridiska personer.

Namn: Karolinska Institutet, Institutionen för Klinisk Neurovetenskap, Sektionen för Personskadeprevention

Adress: Berzelius väg 3, 171 77 Stockholm

1:2 Behörig företrädare

Behörig företrädare för forskningshuvudmannen (t.ex. prefekt, enhetschef, verksamhetschef). Forskningshuvudmännen bestämmer själva, genom interna arbets- och delegationsordningar eller genom fullmakt, vem som är behörig att företräda forskningshuvudmannen. Bifoga kopia av sådan handling.

.

Namn: Kristina Alexanderson Tjänstetitel: Professor, Sektionschef

Adress: Karolinska Institutet, Institutionen för Klinisk Neurovetenskap, Sektionen för Personskadeprevention,

Berzelius väg 3, 171 77 Stockholm

1:3 Forskare som är huvudansvarig för genomförandet av projektet (kontaktperson)

Namn: Irene Jensen, professor

Adress: Karolinska Institutet, Institutionen för Klinisk Neurovetenskap,
Sektionen för Personskadeprevention,
Berzelius väg 3, 171 77 Stockholm

E-postadress: irene.jensen@ki.se

Telefon: 08 524 83 212

Mobiltelefon: 070 577 76 59

1:4 Plats

Plats/er där projektet skall genomföras (ange inrättning/ar, institution/er, klinik/er etc.).

Sektionen för Personskadeprevention, Institutionen för Klinisk Neurovetenskap, Karolinska Institutet. Datainsamling sker på vårdmottagningar i hela Sverige där legitimerade kiropraktorer är verksamma.

1:5 Andra medverkande

Övriga deltagande forskningshuvudmän samt forskare ansvariga att lokalt genomföra projektet (kontaktpersoner) skall anges i bilaga (namn, adresser).

1:6 Vid läkemedelsprövning

Ansökan om tillstånd har insänts till Läkemedelsverket.

Ansökan inlämnad (datum):

Tillstånd erhållits ☐

1:7 Vid viss genetisk forskning

Anmälan till *Datainspektionen* om förhandskontroll av behandling av personuppgifter om genetiska anlag som framkommit efter genetisk undersökning (10 § första stycket 2 personuppgiftsförordningen (1998:1191).

Inlämnad (datum): Kommer att inlämnas efter godkänd etikprövning ☐

2. Uppgifter om projektet

2:1 Sammanfattande beskrivning av forskningsprojektet (programmet)

Beskrivningen skall kunna förstås av nämndens lekmän. Undvik därför terminologi som kräver specialkunskaper. Ange bakgrund och syfte för studien samt den (de) vetenskapliga frågeställning (ar) som man söker svar på.
Ange de viktigaste undersökningsvariablerna. Ange vilka kunskapsvinster projektet kan förväntas ge och betydelsen av dessa. Ange om det är en registerstudie, uppdragsforskning etc.
För fackmän avsedd detaljerad information i protokoll eller forskningsplan *skall* bifogas som bilaga. En utförligare beskrivning över genomförandet *avsedd för lekmän* kan vid behov bifogas den för fackmän avsedda obligatoriska forskningsplanen.

Ländryggssmärta är ett folkhälsoprobelm i Sverige och i Väst-världen. Förekomsten är hög och kostnaden för individen och samhället likaså. Ländryggssmärta är dessutom ofta ett återkommande problem för den som drabbas, varför kunskap om preventiva åtgärder behövs.
Kiropraktorer behandlar traditionellt ryggsmärta, och numera föreligger evidens angående metodens effektivitet vid kortvarig behandling. Det finns även en lång tradition inom professionen att behandla långsiktigt förebyggande, men detta har hittills ej testats vetenskapligt.

Inledande studier har definierat patienten som kiropraktorerna anser lämplig för sådan behandling.Nästa steg är nu att följa en grupp av dessa patienter för att se i vilken grad de besväras av sin ländryggssmärta (delstudie1), för att slutligen testa metodens effektivitet (delstudie 2).
Syftet med den första delstudien är att definiera patienternas profiler avseende besvärsgrad, sjukskrivning och behandling. Detta för att, i andra delstudien, kunna se om regelbunden kiropraktisk behandling kan förändra till det bättre samt huruvida detta är ett kostnadseffektivt sätt att behandla.

2:2 Vilken primär vetenskaplig frågeställning ligger till grund för projektets utformning

Om projektet kan karakteriseras som en hypotesprövning, ange den primära och eventuellt sekundära hypotesen. Hänvisning till mer detaljerad information för fackmän kan ske till bifogat protokoll eller forskningsplan enligt 2:1

1: Hur varierar ländryggspatienternas smärta under sex månader, i relation till besvärsgrad och sjukfrånvaro?
2: Är det möjligt att definiera under-grupper i relation till besvärsgrad?
3: Hur varierar ländryggssmärtan under tolv månader (besvärsgrad, sjukfrånvaro, självskattad hälsa) hos patienter som får regelbunden kiropraktisk behandling jämfört med dem som får behandling bara vid akut smärta?
4: Är preventiv behandling kostnadseffektiv?

2:3 Redogör för resultat från relevanta djurförsök

För viss, främst medicinsk, forskning ange skälen till att djurförsök inte utförts.

Ej relevant

2:4 Redogör översiktligt för undersökningsprocedur, datainsamling och datas karaktär

Av beskrivningen skall framgå hur studien planeras genomföras. Beskriv insamlade datas karaktär. Hur säkerställs datas tillförlitlighet (t.ex. kvalitetskontroll/monitorering)? - Vid enkäter och intervjuer skall beskrivas tillvägagångssätt och t.ex. frågors innehåll och hur slutsatser dras. Enkäter och skattningsskalor skall bifogas.<br>- För medicinsk forskning skall anges t.ex. typer av ingrepp, mätmetoder, antal besök, tidsåtgång vid varje försök, doser och administrationssätt för eventuella läkemedel och/eller isotoper, blodprovsmängd (även ackumulerad mängd vid multipla försök). Ange även om och på vilket sätt undersökningsprocedur m.m. skiljer sig från klinisk rutin. Ange proceduren för att ge den eventuella behandling efter studiens slut, som kan erfordras. Ange procedur för insamling av biologiskt material. Redogör för datakällor och procedurer vid behandling av personuppgifter. För mer detaljerad information kan hänvisning ske till bilagt protokoll eller forskningsplan enligt 2:1.

Patienter som söker vård hos en kiropraktor för ländryggssmärta, kommer att tillfrågas om att medverka i första delstudien.Vid andra och fjärde konsultationen fylls en enkät i av kiropraktorn tillsammans med patienten. Patienten får också fylla i en enkät själv vid andra konsultationen. Efter sex månader skickas en enkät till patienten. Enkäterna innehåller dels beskriptiv data (kön, ålder, typ av besvär, duration och frekvens), dels data om självskattad hälsa och efterhand förbättring. Frågeformulären är validerade i tidigare undersökningar. Patienten får skriftlig information och får fylla i en medgivande blankett om han/hon väljer att delta.
Under sex månader får patienten ett SMS varje vecka med fråga om besvärsgrad. Månadsvis kommer även en SMS-fråga om sjukfrånvaro samt en om behandling.
SMS- svaren registreras av ett datorsystem, SMS Track, som gör profiler av varje patient. Enkätsvaren läses in optiskt och svaren läggs i databasen med ett löpnummer och utan personuppgifter. All persondata kopplas till ett löpnummer, och "nyckeln" förstörs efter avslutad insamling. Ingen intervention görs i den första delstudien, det är en observationsstudie. Patienterna kommer att vara i en helt normal klinisk situation, där behandlande kiropraktor kommer att ge den behandling som anses nödvändig för patienten.
I den andra delstudien rekryteras patienter på samma sätt. Samma enkäter kommer att fyllas i som delstudie 1 vid samma intervall samt efter 12 månader.Samma data kommer att inhämtas från patient och kiropraktor, och patientens medgivande kommer att sökas på samma sätt. Patienten följs upp av SMS Track-programmet under 12 månader, datan hanteras på samma sätt. Hälften av patienterna kommer att behandlas regelbundet av "sin" behandlande kiropraktor, hälften kommer att få behandling endast när de söker

med akut smärta. Även detta speglar den klinska vardagen, då vissa kiropraktorer behandlar regelbundet och andra endast vid akut smärta.

2:5 Redogör för om insamlat biologiskt material kommer att förvaras i en biobank

Med biobank avses biologiskt material från en eller flera människor som samlas och bevaras tills vidare eller för en bestämd tid och vars ursprung kan härledas till den eller de människor från vilka materialet härrör.

Redogör för var och hur prover som skall sparas förvaras, kodningsprocedurer och villkor för utlämnande av prover. Observera att i förekommande fall skall anmälan av biobank ske till Socialstyrelsen enligt lagen (2002:297) om biobanker i hälso- och sjukvården m.m.

Ej relevant

2:6 Redovisa tillgång till nödvändiga resurser under hela projektets genomförande

Ange vilka som har ansvaret (prefekt, verksamhetschef eller motsvarande) för forskningspersonernas säkerhet vid alla enheter/kliniker där patienter ingår samt att erforderliga ekonomiska och personella resurser finns tillgängliga. Intyg från dessa skall bifogas.

Ansvaret för patienternas fysiska säkerhet ligger som alltid på behandlande kiropraktor, vilka samtliga har patientförsäkringar hos Salus- Ansvar.
Ansvaret för hantering och lagring av data har professor Irene Jensen.
Medel till inköp av SMS Trackprogrammet samt för att ersätta patienternas SMS-kostnader finns hos Legitimerade Kiropraktorers Riksorganisation, LKR.
Professor Irene Jensen är avlönad av Karolinska Institutet.
Till medverkande kiropraktorer utgår ingen ersättning, och patienterna betalar behandlingsarvodet som vanligt.

2:7 Journalföring, registrering och hantering av data

Redogör för hur undersökningsprocedurer och eventuella ingrepp journalförs. Ange hur registrering och behandling av resultaten skall gå till. Om materialet skall kodas, ange proceduren, vem som förvarar kodlistor och vem eller vilka som har tillgång till dem, var de förvaras, hur länge samt om materialet kommer att anonymiseras eller förstöras. Används band- och videoinspelningar? Vilken tillgänglighet har datamaterialet? Hur förvaras det? Hur erhålls erforderligt sekretesskydd?

Kiropraktorerna för behandlingsjournal enligt journalföringslagen, ingen anteckning behöver göras om patientens medverkan i studien eftersom studien inte avviker från normal klinisk praxis.
Patienternas medverkan kommer att vara anonym vid analysen, då de identifieras med ett löpnummer. Endast den ansvariga forskaren har tillgång till listorna med löpnummer, vilka kommer att förvaras i låst och brandsäker förvaring. Detta materialet kommer att förstöras när dataanalysen är genomförd.

2:8 Redogör för tidigare erfarenheter (egna och/eller andras) av den använda proceduren, tekniken eller behandlingen

Särskilt angeläget är att redovisning av risker för komplikationer görs tydliga och i förekommande fall med angivande av relevanta publikationer. Om ansökan avser fortsättning eller uppföljning av tidigare projekt, ange diarienummer samt datum för beslut av tidigare godkänd ansökan. Vid nya läkemedelsbehandlingar av patienter bör anges hur många patienter (med aktuell eller annan åkomma) som tidigare erhållit föreslagen eller högre dosering samt hur långa behandlingsperioder som studerats.

Detta är den första studien av sitt slag i Sverige. Med forskare i Danmark och Finland pågår ett samarbete där flera studier tar upp olika aspekter av frågeställningar kring preventiv kiropraktisk behandling.

3. Uppgifter om forskningspersoner

3:1 Hur görs urvalet av forskningspersoner

Med forskningsperson avses en levande människa som forskningen avser.

Ange urvalskriterier (inklusion och exklusion). På vilket sätt kommer forskaren i kontakt med/får kännedom om lämpliga forskningspersoner? Ange om rekrytering sker från (egna, andras) tidigare eller pågående studier. Om annonsering sker, skall annonsmaterialet insändas som bilaga. Om t.ex. barn, eller personer som tillfälligt eller permanent inte är kapabla att ge ett eget informerat samtycke skall tillfrågas om deltagande i projektet, skall detta särskilt motiveras. Om vissa grupper (t.ex. kvinnor, barn eller äldre) utesluts från deltagande i projektet skall detta särskilt motiveras.

Inklusionskriterier för kiropraktorer: 1) Medlem i LKR, vilket garanterar en godkänd akademisk utbildning. 2) Villiga att hjälpa till med att samla in data. 3) Har, i en tidigare studie, sagt sig tro att kiropraktisk behandling kan förebygga återfall av ländryggssmärta.
Patienterna ska 1) vara mellan 18-65 år (arbetsför ålder) och 2) söka kiropraktor för återkommande ländryggssmärta sedan 1-2 år. De ska 3) inte redan vara kiropraktorpatienter på regelbunden behandling, 4) gravida uteslutas och patienterna 5) måste kunna förstå svenska språket, 6) måste ha en egen mobiltelefon och 7) behärska SMS-funktionen och slutligen 8) vara villiga att delta i studien.

3:2 Ange relationen mellan forskare/försöksledare och forskningspersonerna

☐ Behandlare (t.ex. läkare, psykolog, sjukgymnast) - forskningsperson (t.ex. patient, klient)

☐ Kursgivare (lärare) - student

☐ Arbetsgivare - anställd

☒ Annan relation. Beskriv: Ansvarig forskare kommer aldrig själv att träffa forskningspersonerna.

3:3 Redogör för det statistiska underlaget för studiepopulationens (ernas)/ undersökningsmaterialets(-ens) storlek

Redovisa en statistisk styrka, så kallad ”power”-beräkning eller motsvarande överväganden för tydliggörande av studiens möjligheter att besvara frågeställningarna.

40 kiropraktorer i varje delstudie samlar data under 3 månader, på upp till 10 patienter vardera. Således kommer upp till 400 patienter att ingå i varje delstudie. Det beräknas vara tillräckligt antal för att upptäcka skillnader i utfallsparametern även om en viss andel (10-20%) avbryter deltagandet.
SMS Track är en relativt ny metod där inga tidigare data på denna patientgrupp finns tillgängliga för att möjliggöra en powerberäkning.

3:4 Kan forskningspersonerna komma att inkluderas i flera studier samtidigt eller i nära anslutning till denna studie

Ange om forskningspersonerna kan inkluderas samtidigt i flera studier eller i nära anslutning till denna studie. Ange i så fall projekttitel, forskningshuvudman, forskare som genomför studien (kontaktperson) samt diarienummer (om känt) för de övriga studierna. När avslutades ett eventuellt tidigare deltagande?

I kommande studier börjar vi "från början" igen, och kommer att använda en ny grupp kiropaktorer. Det samma kommer att gälla då: att patienten inte redan är en kiropraktorpatient på regelbunden behandling.

3:5 Vilket försäkringsskydd finns för de forskningspersoner som deltar i projektet

Det åligger forskningshuvudmannen att kontrollera att befintliga försäkringar täcker eventuella skador som kan uppkomma.

Samtliga kiropraktorer är ansvarsförsäkrade i Salus Ansvar genom Legitimerade Kiropraktorers Riksorganisation, LKR.

3:6 Vilken ekonomisk ersättning eller andra förmåner utgår till de forskningspersoner som deltar i projektet och när betalas ersättningen ut (Utförligare beskrivning kan lämnas i bilaga)

Ersättning för obehag och besvär. Belopp (före skatt):

Ersättning för förlorad arbetsinkomst ☐ Ja ☒ Nej

Reseersättning ☐ Ja ☒ Nej

Befrielse från kostnader för läkemedel ☐ Ja ☒ Nej

Befrielse från andra kostnader. Vilka? Kostnader för SMS kommer att ersättas.

Andra förmåner. Vilka?

När betalas ersättningen ut? Kontant i samband med sista enkäten.

Ingen ersättning betalas ut ☐

4. Information och samtycke

4:1 Proceduren för och innehållet i den *information* som lämnas då forskningspersoner tillfrågas om deltagande

Beskriv hur och när information ges och vad den innehåller. Vem informerar? Normalt skall en kortfattad och lättförståelig skriftlig information ges. Denna skriftliga information skall bifogas ansökan. Om ingen eller ofullständig information ges, måste skälen för detta noggrant anges.

Forskningspersonen skall informeras om

- den övergripande planen för forskningen
- syftet med forskningen
- de metoder som kommer att användas
- de följder och risker som forskningen kan medföra
- vem som är forskningshuvudman och kontaktperson
- att deltagandet i forskningen är frivilligt och
- forskningspersonens rätt att när som helst avbryta sin medverkan.

När försökspersonen, patienten, tillfrågas om deltagande, dvs vid andra konsultationen, får vederbörande ett informationsblad där syftet med undersökingen och omfattningen av deltagandet är beskrivet.Om patienten samtycker, får han/hon fylla i en medgivandeblankett. Se bilaga.
Kiropraktorerna som kommer att medverka till datainsamling i studien har, i en tidigare studie, skriftligen sagt sig villiga att delta i en studie om preventiv kiropraktisk behandling.

4:2 Hur och från vem inhämtas ***samtycke***

Beskriv proceduren; vem som frågar, när detta sker och hur samtycket dokumenteras. Utförlig redovisning är särskilt viktig då barn eller personer med nedsatt beslutskompetens ingår i studien, likaså vid studier av en grupp (grupper), t.ex. föreningar, organisationer, företag, kyrkosamfund och församlingar eller arbetet i en skolklass.

Den behandlande kiropraktorn frågar patienten vid andra konsultationen om medverkan, varpå patienten ger sitt skriftliga samtycke innan deltagande i studien i enlighet med de tidigare definierade kriterierna.

5. Forskningsetiska överväganden

5:1 Redogör för risker som deltagandet kan medföra samt möjliga komplikationer

Detta kan vara t.ex. smärta, obehag eller integritetsintrång som projektet innebär eller kan innebära.
Har åtgärder vidtagits för att förebygga de risker som sägs ovan? Vilken beredskap finns att hantera dessa komplikationer? Ange metoder som kommer att användas för att efterforska, registrera och rapportera oönskade händelser.

Denna studie kommer ej att avvika från den kliniska verkligheten behandlingsmässigt. Patienten får precis den vård som den behandlande kiropraktorn anser behövs vare sig de deltar i studien eller ej. Det deltagande patienter får, är ett SMS varje vecka (+ två månadsvis) där han/hon blir tillfrågad om ryggens besvärsgrad. Om något, kan detta sätta fokus på ryggproblemet för patienten.
Patienten får tillfälle att rapportera om eventuella obehag efter behandling vid flera tillfällen i möte med behandlande kiropraktor, se punkt 2:4. Patientens medverkan är anonym enligt punkt 2:7.

5:2 Redogör för förutsebar nytta för de forskningspersoner som ingår i projektet

Eftersom studien speglar den kliniska vardagen, har försökspersonerna ingen direkt nytta av att delta utöver nyttan av behandlingen de får, vilken de skulle fått även utanför studien. Den generella nyttan för alla med ryggbesvär är att ytterligare kunskap erhålles om vilka behandlingar som är effektiva, även preventivt.

5:3 Gör en egen värdering av förhållandet risk – nytta för de forskningspersoner som deltar

Då studien inte avviker från den klinska vardagen, innebär den liten risk för patienterna, kiropraktisk behandling är en relativt säker metod med få biverkningar. Nyttan kan däremot bli betydligt viktigare, eftersom vi kommer att få kunskap om huruvida regelbunden kiropraktisk behandling är effektivt för patienter med återkommande ländryggssmärta .

5:4 Identifiera och precisera vilka etiska problem t.ex. risk – nytta i ett vidare perspektiv som kan uppstå inom eller genom projektet

Det finns en viss risk att patienterna "sjukliggörs" genom att de varje vecka påminns om sin ryggåkomma. De behandlande kiropraktorerna kan också tänkas anstränga sig mer än vanligt för att kunna hjälpa de berörda patienterna.

6. Redovisning av resultaten

6:1 Hur garanteras forskningshuvudmannen och medverkande forskare tillgång till data (anges vid t.ex. uppdragsforskning) och vem ansvarar för databearbetning och rapportskrivning

Vid uppdragsforskning anges hur forskningshuvudmannen och medverkande forskare garanteras tillgång till data och vem som ansvarar för databearbetning och rapportskrivning.

Forskningen äger rum vid Karolinska Institutet och Irene Jensen har således tillgång till datan direkt och ansvarar för databearbetning och rapportskrivning.

6:2 Hur kommer resultaten att göras offentligt tillgängliga
Kommer studien att insändas för publicering i tidskrift eller publiceras på annat sätt

Ange i vilken form resultaten planeras offentliggöras samt tidsplan för detta.

En forskningsartikel planeras för varje delstudie i en vetenskaplig tidskrift, studien är en del av en doktorsavhandling. Publicering beräknas till 2009/2010.

6.3 På vilket sätt garanteras forskningspersonernas rätt till integritet när materialet offentliggörs/publiceras

Beskriv procedurer eller metod för avidentifiering/anonymisering. Redovisas endast resultat på statistisk gruppnivå?

All rapportering sker anonymt och på gruppnivå, vare sig kiropraktorernas eller patienternas identiteter kommer att vara synliga när dataanalys och rapportskrivning ska genomföras. Efter avslutad studie kommer kopplingen mellan patienternas löpnummer och namn att förstöras enligt punkt 2:7.

7. Redovisning av ekonomiska förhållanden och beroendeförhållanden

7:1 Vid uppdragsforskning

Ange uppdragsgivaren t.ex. vid klinisk läkemedelsprövning.

Namn: Ej relevant

Kontaktperson:

Adress:

Telefon/mobiltelefon:

Ange uppdragsgivarens relation till forskningshuvudmannen/medverkande forskare, t.ex. anställningsförhållande.

7:2 Redovisa eventuella ekonomiska överenskommelser med uppdragsgivare eller andra finansiärer (namn, belopp)

Vid klinisk läkemedelsprövning kan hänvisning ske till ingånget avtal med sjukvårdshuvudmannen eller genom uppgift om föreslagen ersättning enligt överenskommelsen mellan Landstingsförbundet och LIF som bifogas. Separata överenskommelser med den/de som skall genomföra forskningen skall också redovisas. Om överenskommelserna inte är klara i sin helhet vid tidpunkten för ansökan skall belopp för studien/ersättning till kliniken/genomföraren och vad ersättningen skall täcka alternativt belopp per forskningsperson anges här. Vid studier där fler än en forskningshuvudman deltar skall principerna för och storleksordningen för ersättning för studien i sin helhet anges.

Ej relevant

7:3 Redovisa forskningshuvudmannens och medverkande forskares intressen/tillgångar

Redovisa de som kan tänkas påverka tilltron till objektiviteten i genomförande och rapportering (t.ex. aktieinnehav eller konsultuppdrag i finansierande företag).

Ej relevant

8. Förteckning över bilagor

Dokument som, i tillämpliga fall, skall bifogas om inte motsvarande information finns i blanketten har markerats med x. Markera de bilagor som skickas in med denna ansökan.

Insänd med ansökan	Bil nr	Beskrivning	Klinisk läkemedels prövning	Annan forskning
☐	1<br>p 1:5	Deltagande forskningshuvudmän och medverkande forskare (kontaktpersoner) vid forskning där mer än en forskningshuvudman deltar	x	x
☒	2<br>p 2:1	För fackmän avsedd projekt/forskningsplan (protokoll), vid behov även för lekmän avsedd bilaga	x	x
☐	3<br>p 3:1	Annonsmaterial för rekrytering av forskningspersoner	x	x
☒	4<br>p 4:1	Skriftlig information till dem som tillfrågas	x	x
☒	5<br>p 2:4	Enkät, frågeformulär	x	x
☐	6	Gemensam EU-blankett (gäller fr.o.m. den 1 maj 2004), gäller även vid ändring	x	
☐	7	Sammanfattning av protokollet på svenska	x	
☐	8	Prövarhandbok alt. bipacksedel/produktresumé	x	
☒	9<br>p 2:6	Intyg från verksamhetschef/motsv. om resurser	x	x
☒	10	CV för forskare (samma som p 1:3) med huvudansvar för genomförande (redovisa forskarens kompetens)	x	x
☐	11<br>p 3:6	Beskrivning av ersättning till forskningspersoner	x	x
☐	12<br>p 7:1<br>p 7:2	Överenskommelser med uppdragsgivare/finansiär om t.ex. anställningsförhållanden, bidrag/ersättning till prövningsplats, sjukvårdshuvudman, forskningshuvudman eller forskare	x	x
☐	13	Tillstånd från strålskyddskommitté eller motsvarande	x	x

9. Undertecknande

Behörig företrädare för sökande forskningshuvudman enligt p 1:2.

Ort: Stockholm Datum: 20/11 2007

Signatur

Namnförtydligande: Kristina Alexanderson

Undertecknad forskare som genomför projektet (kontaktperson) enligt p 1:3 intygar härmed att forskningen kommer att genomföras i enlighet med ansökan.

Ort: Stockholm Datum: 20/11 2007

Signatur

Namnförtydligande: Irene Jensen

Anvisning för ansökan

Denna ansökningsblankett används vid ansökan om etikprövning enligt lagen (2003:460) om etikprövning av forskning som avser människor. Den är avsedd att användas för all slags forskning där godkännande skall inhämtas från en etikprövningsnämnd. Blanketten skall användas även vid begäran om rådgivande yttrande enligt 2 § förordning (2003:616) med instruktion för regionala etikprövningsnämnder. Beroende på vilken forskning som ansökan gäller kommer de uppgifter som efterfrågas nedan att ha olika relevans. Därmed varierar också kravet på utförlighet i redovisningen av dessa. Markera på formuläret när uppgiften inte berör det aktuella projektet. Ansökan skall ifyllas så att den blir lättläst, dvs. den skall inte vara handskriven och inte skriven med liten stil och kort radavstånd. Blanketten skall skrivas på svenska.

Observera att en ansökan aldrig är komplett (och därmed kan behandlas) förrän blanketten är korrekt ifylld och avgiften är betald.
Ansökan med bilagor insändes i ett original och 16 kopior!

Om denna blankett
Allt som skall fyllas i är markerat grått. Övrig förklarande text är låst och får inte ändras. De grå fälten expanderar allteftersom du skriver, det finns alltså obegränsat utrymme för din text. Vid utskrift syns enbart texten du fyllt i och ej de grå fälten som syns på skärmen, det är därför viktigt att kontrollera att allt verkligen är ifyllt. När du är klar skriver du ut blanketten, kompletterar med erforderliga underskrifter samt bilagor och skickar till den regionala etikprövningsnämnd till vars upptagningsområde forskningshuvudmannen hör, se www.forskningsetikprovning.se.

EPN
REGIONALA ETIKPRÖVNINGSNÄMNDEN
STOCKHOLM

FÖR KÄNNEDOM

PROTOKOLL 2008/4:1
2008-01-09

Sammanträde i Stockholm

Avdelning 4

NÄRVARANDE

Ledamöter
Annika Marcus, ordförande
Pierre Lafolie, vetenskaplig sekreterare (*klinisk farmakologi*)
Maria Kumlin (*medicin, astmasjukdomar)*
Gunnar Johanson, *(toxikologi)*
Guro Gafvelin (*molekylär immunologi*)
Erling Löfsjögård Nilsson (*klinisk fysiologi)*
Erik Näslund (*kirurgi*)
Anna-Berit Ransjö Arvidsson (*omvårdnad)*
Roland Sennerstam (*pediatrik*)
André Stark *(ortopedi)*
Erik Sundström (*geriatrik)*
Elisabeth Fleetwood (*allmänföreträdare*)
Roland Håkansson (*allmänföreträdare)*
Benkt Kullgard (*allmänföreträdare), deltar inte i ärende 2007/1384*
Åke Reisnert ((*allmänföreträdare))*
Gunnar Skoglund (*allmänföreträdare)*

Övriga
Lena Creutzer Waldersten, *administrativ sekreterare*

---

§ 1
Ordföranden förklarar sammanträdet öppnat.

§ 2
Ansökningar om etisk granskning av forskningsprojekt, se **Bilaga.**

§ 3
Ordföranden meddelar att nästa sammanträde i avdelning 4 äger rum den 6 februari 2008.

§ 4
Ordföranden förklarar sammanträdet avslutat.

Stockholm som ovan

Annika Marcus
Ordförande

Pierre Lafolie
Vetenskaplig sekreterare

Adress	Besöksadress	Telefon	Fax	E-post	Hemsida
Box 289	Nobels väg 12 A	08-52480000 (vxl)	08-524 86699	kansli@stockholm.epn.se	www.epn.se
171 77 Stockholm	Solna	08-52486472 (dir)		lena.creutzer.waldersten@ki.se	

---Utdrag ur protokoll --

Diarienummer och
Föredragande:

2007/1458-31/4
André Stark

Sökande: Karolinska Institutet
Behörig företrädare: Kristina Alexandersson
Projekt: Kiropraktisk behandling vid återkommande ländryggssmärta.
Forskare som genomför projektet: Irene Jensen

BESLUT

Projektet avser inhämtande av känsliga personuppgifter, men med uttryckligt samtycke därtill. Projektet är därmed inte tillståndspliktigt enligt bestämmelserna i lagen (2003:460) om etikprövning av forskning som avser människor.

Nämnden lämnar med stöd av 2 § förordningen (2003:616) med instruktion för regionala etikprövningsnämnder följande

RÅDGIVANDE YTTRANDE

Nämnden har inga invändningar mot projektets genomförande även om sökanden bör överväga införandet av en kontrollgrupp samt randomisering för att öka det vetenskapliga värdet.

Hur man överklagar beslutet att forskningen inte omfattas av etikprövningslagen, se särskild information.

Det rådgivande yttrandet får inte överklagas.

Att den avskrift i transumt överensstämmer med originalet intygar:

Lena Waldersten

Lena Creutzer Waldersten, administrativ sekreterare

/Expedierat 2008-01-18/

Regionala etikprövningsnämnden i Stockholm
Protokoll 2008/4:1

---Utdrag ur protokoll --

Diarienummer och
Föredragande:

2007/1458-31/4
André Stark

Sökande: Karolinska Institutet
Behörig företrädare: Kristina Alexandersson
Projekt: Kiropraktisk behandling vid återkommande ländryggssmärta.
Forskare som genomför projektet: Irene Jensen

BESLUT

Projektet avser inhämtande av känsliga personuppgifter, men med uttryckligt samtycke därtill. Projektet är därmed inte tillståndspliktigt enligt bestämmelserna i lagen (2003:460) om etikprövning av forskning som avser människor.

Nämnden lämnar med stöd av 2 § förordningen (2003:616) med instruktion för regionala etikprövningsnämnder följande

RÅDGIVANDE YTTRANDE

Nämnden har inga invändningar mot projektets genomförande även om sökanden bör överväga införandet av en kontrollgrupp samt randomisering för att öka det vetenskapliga värdet.

Hur man överklagar beslutet att forskningen inte omfattas av etikprövningslagen, se särskild information.

Det rådgivande yttrandet får inte överklagas.

Att den avskrift i transumt överensstämmer med originalet intygar:

Lena Waldersten

Lena Creutzer Waldersten, administrativ sekreterare

/Expedierat 2008-01-18/
