# Supplementary material for: The Nordic Maintenance Care program: Effectiveness of chiropractic maintenance care versus symptom-guided treatment for recurrent and persistent low back pain—A pragmatic randomized controlled trial
Source: PLoS One. 2018 Sep 12;13(9):e0203029. doi: 10.1371/journal.pone.0203029 (PMC6135505; doi:10.1371/journal.pone.0203029)
Supplement: S2 File — (PDF) [file pone.0203029.s002.pdf]

S2 File

English translation of the key methodological portions of the original trial protocol part of the ethical application (participant inclusion/exclusion criteria, descriptions of the intervention and outcomes).

Author comments, July 20, 2018:

As required by the editorial office this translation only concerns the key methodological portions of the protocol that was submitted for the ethical application (November 20, 2007) and not the complete document. As suggested by the ethical review board changes were made to the protocol after the ethical application was approved. Some of these changes where to include a control group and random allocation of subjects in the 2nd study. For a detailed description of the study that was conducted please refer to the published study protocol (**S1_File_ Study_Protocol**).

Eklund A, Axen I, Kongsted A, Lohela-Karlsson M, Leboeuf-Yde C, Jensen I. Prevention of low back pain: effect, cost-effectiveness, and cost-utility of maintenance care - study protocol for a randomized clinical trial. Trials. 2014;15(1):102.

Translation of key methodological portions:

1:4 Location:

Sektionen för Personskadeprevention, Institutionen för Klinisk Neurovetenskap, Karolinska Institutet. Data collection will be conducted in clinics, where licensed Chiropractors are active, across Sweden.

2:2 Which primary scientific question forms the basis of the outline of the project:

1. How does low back pain vary, in relation to symptom severity and sick leave, during 6 months.
2. Is it possible to identify sub-groups in relation to symptom severity?
3. How does low back pain vary during 12 months (symptom severity, sick leave, self-rated health) among patients who receive regular chiropractic treatment compared to those who only get treatment in response to acute pain.
4. Is preventive treatment cost effective.

2:4 In general, describe examination procedure, data collection and type of data.

Patients seeking care from chiropractors for low back pain, will be asked to participate in the 1st study of the project. At both at the 2nd and 4th consultation, the chiropractor together with the patient fill out a questionnaire. At the second consultation, the patient is also given a questionnaire to fill in. After 6 months a questionnaire is sent to the patient. The questionnaires contains descriptive data (sex, age, type of condition, duration, frequency) and data on self-rated health and self-rated improvement (at the 4th visit). The questionnaires are validated in previous studies. The

patient is given written information regarding the trial and a consent form to fill in to declare if he/she wish to participate. During 6 months the patient receives an SMS each week asking for the status of their condition. Monthly two additional SMS-questions will be sent, one regarding sick leave and one regarding the treatments. The SMS-replies are collected by an automated computer system, SMS-Track, that makes profiles of the data on each patient.
The data from the questionnaires will be added to the database with an optical reader and an ID-number will replace all personal data. All personal data is linked to the ID-number using a key that will be destroyed after the data collection is completed.
The first study is an observational study and will not contain a specific intervention. Patients will be observed in a normal clinical situation, where the treating chiropractor will administer the most appropriate treatment for each patient.
In the 2nd study of the project patients will be recruited in the same way. The same questionnaires at the same interval will be included, as well as adding a measurement at 12 months. The same data will be collected from patients and chiropractors, including an informed consent form. Patients will be followed with the SMS-track system during 12 months, the data will be handled in the same way as in the 1st study. Half of the patients will be treated regularly by "their" chiropractor, and half will only receive treatment when they present with acute pain. These two strategies represents normal clinical procedures, as some chiropractors treat patients regularly irrespectively of pain status and some only when they present with acute pain.

3:1 How are study subjects selected?

Inclusion criteria for chiropractors: 1) Member of SCA (Swedish Chiropractic Association), which ensures an accredited academic education. 2) Willing to help collect patient data. 3) Has, in a previous study, communicated a belief that chiropractic care may prevent recurrent episodes of low back pain.

Inclusion criteria for patients: 1) To be of working age (18-65). 2) Consulting a chiropractor for recurrent low back pain of 1-2 years duration. 3) Not already a chiropractic patient under maintenance care. 4) Not pregnant. 5) Understand Swedish. 6) Have access to a mobile phone. 7) Be able to send and receive SMS. 8) To be willing to participate in the study.

3:3 Describe the statistical basis for the study population/material size.

40 chiropractors in each study collect data during 3 months, up to 10 patients each. Thus, 400 patients will be included in each study. It is estimated to be enough study subjects to detect a difference in the primary outcome measure even with a 10-20% dropout rate. SMS-Track is a relatively novel method where no previous data on this patient group is available to enable a statistical power calculation.
