# Supplementary figures and images for: The Nordic Maintenance Care program: Effectiveness of chiropractic maintenance care versus symptom-guided treatment for recurrent and persistent low back pain—A pragmatic randomized controlled trial
Source: PLoS One. 2018 Sep 12;13(9):e0203029. doi: 10.1371/journal.pone.0203029 (PMC6135505; doi:10.1371/journal.pone.0203029)

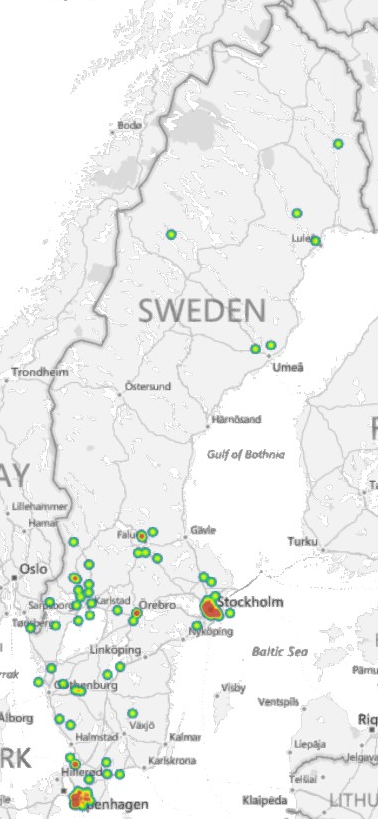

Supplement: S1 Fig — Red zones represents the highest numbers of requited subjects followed by yellow, green and blue. (TIF) [file pone.0203029.s004.tif]
