# Supplementary material for: The Nordic Maintenance Care program: Effectiveness of chiropractic maintenance care versus symptom-guided treatment for recurrent and persistent low back pain—A pragmatic randomized controlled trial
Source: PLoS One. 2018 Sep 12;13(9):e0203029. doi: 10.1371/journal.pone.0203029 (PMC6135505; doi:10.1371/journal.pone.0203029)
Supplement: S1 Table — LBP, Low Back Pain; A, Inclusion criteria; B, Exclusion criteria; MPI, West-Haven Yale Multidimensional Pain Inventory; AC, Adaptive Coper; ID, Interpersonally Distressed; DYS, Dysfunctional; MC, Chiropractic Maintenance Care; RMDQ, Roland Morris Disability Questionnaire; EQ5D, EuroQol 5 dimensions; SD, Standard Deviation. (PDF) [file pone.0203029.s005.pdf]

Variable		1st visit, n=2033	Excluded at 1st visit, n=1122	Lost after 1st visit, n=295	4th visit, n= 616	Excluded at 4st visit, n=176	Lost after 4st visit, n=112	Inclusion visit, n= 328
**1st visit**								
LBP, % (n)		93.9 (1910)	92.1 (1033)	- A	- A	- A	- A	- A
Previous episodes of LBP in recent years, % (n)		74.5 (1514)	57.8 (648)	- A	- A	- A	- A	- A
Previous LBP in total >30 days in past year, % (n)		54.2 (1101)	25.1 (282)	- A	- A	- A	- A	- A
Pain in the thigh, % (n)		21.5 (437)	21.4 (240)	22.0 (65)	21.4 (132)	21.6 (38)	20.5 (23)	21.6 (71)
Pain in the thigh and lower leg, % (n)		18.8 (382)	16.6 (186)	21.4 (63)	21.6 (133)	22.2 (39)	25.9 (29)	19.8 (65)
Pain in the lower leg, % (n)		3.5 (71)	2.7 (30)	4.7 (14)	4.4 (27)	5.7 (10)	6.3 (7)	3.0 (10)
No pain in the leg, % (n)		40.6 (826)	42.2 (474)	39.3 (116)	38.3 (236)	40.3 (71)	33.9 (38)	38.7 (127)
Never visited a chiropractor for this problem before, % (n)		53.4 (1086)	55.4 (622)	49.2 (145)	51.8 (319)	59.7 (105)	50.0 (56)	48.2 (158)
Pain in the neck and/or thoracic spine, % (n)		49.0 (997)	50.5 (492)	63.3 (159)	66.2 (346)	65.2 (105)	61.4 (54)	68.2 (187)
Payment for treatment, % (n)	Completely by patient	90.6 (1672)	91.2 (886)	88.8 (261)	90.5 (525)	89.6 (155)	91.3 (84.8)	90.8 (275)
	Partly by other	9.4 (174)	8.8(86)	11.2 (33)	9.5 (55)	10.4 (18)	8.7 (9)	9.2 (28)

Treatment payed completely by other, % (n)	5.4 (109)	9.4 (105)	- [B]	- [B]	- [B]	- [B]	- [B]
Patient believes that the pain will get better 0-10 (No chance - Very likely), mean (SD)	8.1 (2.1)	8.4 (2.0)	7.5 (2.3)	7.9 (2.1)	7.13 (2.3)	8.0 (1.9)	8.28 (1.9)
Lives alone, % (n)	12.7 (258)	12.5 (140)	14.6 (43)	12.2 (75)	11.9 (21)	8.9 (10)	13.4 (44)
MPI cluster ID / DYS / AC, % (n)	22.2/41.8/36.1 (1566)	19.3/45.2/35.5 (825)	29.2/36.4/34.3 (236)	23.6/38.6/37.8 (505)	20.7/32.0/47.3 (150)	25.3/48.4/26.3 (95)	24.6/38.8/36.5 (260)
Pain severity (MPI) 0-6, mean (SD)	3.4 (1.2)	3.5 (1.3)	3.5 (1.1)	3.3 (1.2)	3.2 (1.3)	3.7 (1.1)	3.3 (1.1)
Interference (MPI) 0-6, mean (SD)	2.9 (1.4)	2.9 (1.4)	2.9 (1.3)	2.8 (1.3)	2.6 (1.3)	3.1 (1.2)	2.8 (1.4)
Life control (MPI) 0-6, mean (SD)	3.5 (1.2)	3.5 (1.2)	3.4 (1.1)	3.5 (1.1)	3.7 (1.0)	3.3 (1.1)	3.5 (1.1)
Affective distress (MPI) 0-6, mean (SD)	2.6 (1.4)	2.5 (1.5)	2.9 (1.4)	2.7 (1.4)	2.6 (1.4)	2.9 (1.3)	2.7 (1.3)
Support (MPI) 0-6, mean (SD)	4.0 (1.7)	4.1 (1.7)	3.7 (1.7)	3.9 (1.7)	3.7 (1.7)	4.1 (1.6)	4.0 (1.7)
Punishing responses (MPI) 0-6, mean (SD)	0.9 (1.1)	0.9 (1.1)	1.1 (1.2)	1.0 (1.2)	0.85 (1.0)	1.1 (1.2)	1.0 (1.3)
Solicitous responses (MPI) 0-6, mean (SD)	2.8 (1.4)	3.0 (1.4)	2.6 (1.4)	2.6 (1.4)	2.5 (1.3)	2.9 (1.5)	2.6 (1.4)
Distracting responses (MPI) 0-6, mean (SD)	2.9 (1.4)	2.9 (1.4)	2.8 (1.4)	2.8 (1.5)	2.8 (1.6)	2.8 (1.4)	2.8 (1.4)
Pain intensity at 1st visit (first measure) 0-10, mean (SD)	5.6 (2.2)	5.7 (2.3)	5.6 (1.4)	5.38 (2.1)	5.24 (2.1)	5.9 (2.0)	5.3 (2.1)
EQ5D score baseline, mean (SD)	0.67 (0.3)	0.66 (0.2)	0.66 (0.2)	0.69 (0.2)	0.70 (0.2)	0.64 (0.2)	0.69 (0.2)
Excellent	8.0 (163)	10.4 (117)	3.4 (10)	5.8 (36)	5.1 (9)	8.9 (10)	5.2 (17)

Health in general (study start), % (n)	Very good	32.1 (653)	33.4 (375)	29.8 (88)	30.8 (190)	35.2 (62)	25.0 (28)	30.5 (100)
	Good	34.6 (704)	31.5 (353)	37.6 (111)	39.0 (240)	39.8 (70)	33.9 (38)	40.2 (132)
	Quite poor	13.3 (271)	10.3 (116)	19.7 (58)	15.7 (97)	13.6 (24)	25.0 (28)	13.7 (45)
	Poor	3.0 (62)	2.5 (28)	4.7 (14)	3.2 (20)	4.0 (7)	1.8 (2)	3.4 (11)
	Missing	8.9 (180)	11.9 (133)	4.7 (14)	5.4 (33)	2.3 (4)	5.4 (6)	7.0 (23)
**4th visit**								
Chiropractor believes that MC is appropriate for patient, % (n)		-	-	-	78.9 (486)	69.3 (122)	81.3 (91)	84.8 (278)
Has taken analgesic medication for the pain, % (n)		-	-	-	18.0 (111)	21.6 (38)	18.8 (21)	15.9 (52)
Pain intensity at 4th visit (second measure) 0-10, n = 246, mean (SD)		-	-	-	3.1 (2.1)	4.3 (2.2)	2.8 (1.9)	2.4 (1.7)
